# Esophageal Perforation Following Pneumatic Dilation in Esophageal Achalasia Successfully Managed with Two-Stage Laparoscopic Surgery

**DOI:** 10.70352/scrj.cr.25-0418

**Published:** 2025-11-29

**Authors:** Yuki Sakai, Eisuke Booka, Ryoma Haneda, Wataru Soneda, Tomohiro Murakami, Tomohiro Matsumoto, Yoshifumi Morita, Hirotoshi Kikuchi, Yoshihiro Hiramatsu, Hiroya Takeuchi

**Affiliations:** 1 Department of Surgery, Hamamatsu University School of Medicine, Hamamatsu, Shizuoka, Japan; 2 Division of Surgical Care, Morimachi, Hamamatsu University School of Medicine, Hamamatsu, Shizuoka, Japan; 3 Department of Perioperative Functioning Care and Support, Hamamatsu University School of Medicine, Hamamatsu, Shizuoka, Japan

**Keywords:** esophageal perforation, esophageal achalasia, two-stage surgery, minimally invasive surgery, Heller–Dor procedure, pneumatic dilation

## Abstract

**INTRODUCTION:**

Endoscopic treatment frequently results in minimal gastrointestinal content leakage and mild symptoms. However, surgical intervention may become necessary when the patient’s general condition is unstable. Further management of the underlying disease is required in cases where esophageal perforation occurs during endoscopic treatment. This report presents a case of esophageal perforation that occurred during the treatment of achalasia, underscoring the success of a two-stage laparoscopic approach.

**CASE PRESENTATION:**

A 72-year-old male with a history of esophageal achalasia underwent treatment. Abdominal pain developed during pneumatic dilation. As the pain intensified, imaging studies revealed esophageal perforation. On admission, his vital signs were consistent with shock, and he was diagnosed with localized esophageal rupture. A two-stage laparoscopic surgery was performed, starting with closure of the 3.5-cm perforation and the creation of a jejunostomy for nutrition. Before the second surgery, the patient received a 10-day enteral nutrition and a laparoscopic Heller–Dor procedure, which was successfully completed without complications. On hospital day 22, the patient was discharged following a stable recovery without perioperative complications.

**CONCLUSIONS:**

This case emphasizes the successful management of esophageal perforation following achalasia treatment through a minimally invasive surgery. The patient underwent curative surgery for esophageal achalasia during a short hospital stay.

## Abbreviations

LHM
: laparoscopic Heller myotomy
PD
: pneumatic dilation
POEM
: per-oral endoscopic myotomy
UGI
: upper gastrointestinal series

## INTRODUCTION

Esophageal perforation can progress to mediastinitis, empyema, severe infection, and multiple organ failure. Therefore, it frequently requires immediate medical intervention.^1)^ Conversely, esophageal perforation following endoscopic treatment typically causes minimal gastrointestinal content leakage. Observational treatment may be considered depending on the severity. Esophageal perforation can occur during achalasia treatment. Surgical intervention for achalasia may be necessary after managing the perforation. However, surgical treatment may become challenging when conservative management is implemented first. Various treatment options exist for esophageal perforation, and recent developments have suggested that less invasive methods can result in better outcomes.^2)^ Therefore, several surgical methods and options are available. We here report a case of esophageal perforation that occurred during esophageal achalasia treatment. After considering several treatment options, a two-stage laparoscopic surgery was conducted. During a relatively short hospital stay, the patient was treated for esophageal perforation and achalasia using only a minimally invasive surgery.

## CASE PRESENTATION

**Fig. 1:**
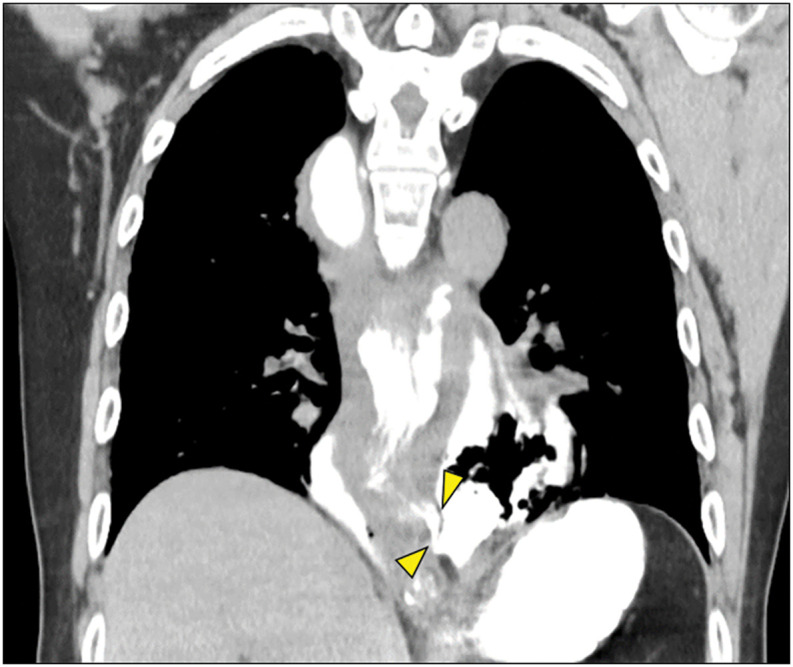
CT imaging. The yellow arrow indicates the perforation site.

The patient was a 72-year-old male with a 14-year history of esophageal achalasia. The degree of dilation was classified as Grade II, and the morphology was diagnosed as advanced sigmoid type.^3)^ Fluoroscopy-guided balloon dilation was performed for managing esophageal achalasia. Following treatment, the patient experienced abdominal pain; therefore, an upper gastrointestinal series was performed. However, no obvious signs of esophageal perforation were detected. As the symptoms worsened over time, a follow-up imaging study was conducted. The patient was diagnosed with esophageal perforation and was immediately referred to our hospital. Upon arrival, he developed hypoxemia and tachycardia. CT revealed leakage of the gastrographin used for gastrointestinal imaging in the lower thoracic esophagus (**Fig. 1**). No obvious pneumothorax or leakage of gastric contents into the mediastinum was observed, leading to the diagnosis of esophageal rupture localized to the mediastinum. The patient’s vital signs were suggestive of shock, and his respiratory condition was unstable. Considering his general condition, we decided to proceed with a two-stage laparoscopic surgery. In other words, after performing surgery for esophageal perforation, we proceeded with surgery for esophageal achalasia.

**Fig. 2:**
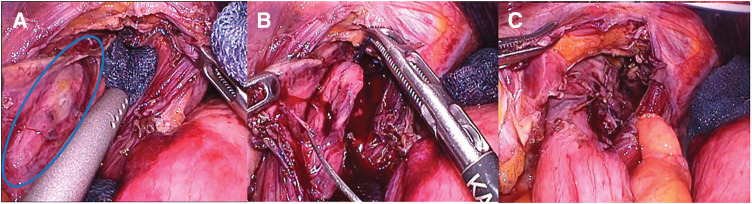
Intraoperative imaging of esophageal perforation. (**A**) The blue circle indicates the perforated area. The long diameter of the hole is 35 mm. (**B**) The perforation is closed by sutures in two layers. (**C**) Completion of the suture.

In the first stage, laparoscopic surgery was initiated to close the perforation site and perform irrigation and drainage. Laparoscopically, the perforation site was confirmed with the assistance of esophagogastroduodenoscopy, revealing an approximately 3.5 cm perforation in the left wall of the lower thoracic esophagus (**Fig. 2A**). Intraoperative findings revealed that the perforation site was not severely inflamed or contaminated, further supporting our decision to proceed with an early second-stage surgery. Mucosal and muscular layer suturing was performed to close the perforation site (**Fig. 2B**), and we determined that the suturing was adequate. The mucosa was sutured with 3-0 Vicryl (Johnson & Johnson K.K., Tokyo, Japan) using simple interrupted stitches, followed by continuous suturing with 3-0 V-Loc (Covidien Japan Inc., Tokyo, Japan). The muscular layer was closed with continuous 3-0 V-Loc sutures. Intraoperative endoscopy with an air leak test confirmed the integrity of the repair. Considering the upcoming second stage of surgery, we decided not to cover the sutured area with the greater omentum or the gastric fundus (**Fig. 2C**). A jejunostomy was created before completing the surgery to manage postoperative nutrition. A 19Fr J-Vac drain (Johnson & Johnson K.K., Tokyo, Japan) was placed on the ventral and dorsal sides of the suture site. The operative time was 4 h and 10 min, and the blood loss was 70 mL.

**Fig. 3:**
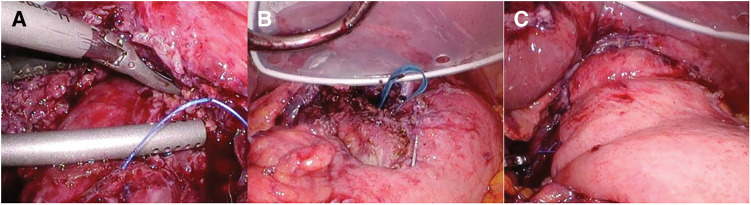
Heller–Dor procedure for esophageal achalasia. (**A**) Adhesion between the sutures and the surrounding tissue. (**B**) Extent of myotomy. (**C**) The gastric fundus is wrapped around the myotomy site.

On POD 2, the patient was transferred out of the ICU and subsequently received 10 days of nutritional management via enteral feeding. Due to the planned short interval before the second surgery, we opted not to initiate oral intake. We observed decreased inflammatory markers on serial blood tests. A CT scan on POD 6 and a gastrografin swallow study on day 7 confirmed no residual perforation. Endoscopy was not performed before the second surgery due to concerns about disrupting the suture site. On POD 11, a laparoscopic Heller–Dor procedure was performed as the second stage of surgery. Additional time was required for careful dissection owing to adhesions between the sutured area and surrounding tissues from the first surgery (**Fig. 3A**). We utilized a combination of blunt dissection and sharp dissection using an ultrasonic coagulation and cutting device, resulting in some damage to the esophageal adventitia during the separation. During intraoperative endoscopy, the esophagogastric junction was confirmed. An incision was made on the anterior wall: 5 cm in the cervical esophagus and 2 cm on the stomach side, cutting through the muscle layer (Supplementary Fig. 1). After creating an additional muscular incision from the esophagus to the gastric wall (**Fig. 3B**), the incision was closed by suturing with coverage from the gastric fundus (**Fig. 3C**). The surgical duration was 5 h and 39 min, and the blood loss was 170 mL.

On POD 3 following the second surgery, oral intake was initiated. The patient was discharged on POD 11 following the second surgery (day 22 of total hospitalization). No perioperative complications were observed.

## DISCUSSION

Esophageal perforation is a serious emergency condition, with a reported mortality rate of 9.1%–21%.^4)^ It is classified as a mediastinal localized type when the mediastinal pleura remains intact and as an intrathoracic type when the mediastinal pleura is ruptured with communication to the thoracic cavity. In the localized type, the esophageal wall is damaged, but the mediastinal pleura stays intact, often resulting in mediastinal or subcutaneous emphysema without pneumothorax.^5,6)^ Conservative management can be suitable if the patient is carefully observed and certain criteria are fulfilled.^5,6)^

Iatrogenic factors, frequently associated with endoscopic procedures, are the most common causes of esophageal perforation.^7)^ However, few consolidated reports on esophageal perforation following endoscopic treatment are available, making it challenging to determine treatment strategies. Before endoscopic procedures, patients are frequently required to have an empty stomach, which may help reduce the risk of contamination. Therefore, some reports have indicated that conservative management with drainage procedures can lead to favorable outcomes.^8)^ Recent advancements in endoscopic procedures have made treatment options, including using clips or stents, viable alternatives.^9)^ However, emergency surgery is occasionally required when the patient’s general condition is unstable, as in the current case.^10)^ In any case, management at a medical facility capable of evaluating treatment strategies with esophageal resection is crucial. Treating underlying diseases like achalasia or cancer must be considered, often requiring staged surgical approaches after initial perforation management.^11)^

Endoscopic pneumatic dilation (PD) and the surgical procedure known as laparoscopic Heller myotomy (LHM) are treatment options for esophageal achalasia. LHM improves symptoms in about 64%, while PD does so in approximately 90%, though perforation rates are 0.51% and 2.0%, respectively.^12)^ A 2023 meta-analysis reports adverse event rates (Clavien–Dindo grade ≥3) of 3.6% for per-oral endoscopic myotomy (POEM), 4.9% for LHM, and 3.1% for PD.^13)^ The three reported cases of treatment following esophageal perforation after esophageal achalasia management are summarized in **Table 1**.^14–16)^ Regarding esophageal perforation, one case underwent emergency surgery using an open abdominal approach to close the perforation site.^14)^ In two cases, conservative management led to improvement.^15,16)^ For the additional treatment of esophageal achalasia, one case underwent staged esophagectomy and reconstruction,^16)^ whereas the other two cases involved only observation of symptoms.^14,15)^

**Table 1:** Reported esophageal perforation cases following pneumatic dilation in esophageal achalasia

Case	Author	Year	Age (years)	Sex	From treatment to onset (h)	Surgical treatment	Oral intake resumed	Hospital stay (days)	Achalasia treatment
1	Yoneyama et al.	2003	56	F	0	+	15 POD	NA	No treatment
2	Lin et al.	2009	48	F	30	−	NA	28	No treatment
3	Asai et al.	2024	76	F	4	−	NA	NA	Two-Stage surgery
4	Present case	2025	72	M	7.5	+	14 POD	23	Heller–Dor procedure

F, female; M, male; NA, not available

In our case, esophageal perforation was successfully managed with a minimally invasive surgery combined with definitive treatment for achalasia. To our knowledge, this is the first report to demonstrate such an approach. The minimally invasive method minimized patient burden, led to fewer complications, and enabled early recovery. Traditional surgery uses thoracotomy for direct access, but minimally invasive techniques provide advantages such as less blood loss and better visualization for secure closure.^17–20)^ Regarding suturing techniques, layer-to-layer closure, if feasible, may potentially facilitate myotomy compared to full-thickness closure. Regarding suture materials, we believe that monofilament absorbable sutures are preferable. If there are concerns regarding the suturing of the perforation site, reinforcement with the omentum or the gastric fundus should also be considered. When patient stability is limited, simpler procedures such as thoracic drainage and gastrostomy can be performed, with staged surgery planned at a later time.^21)^

The present case involved esophageal perforation that occurred during the endoscopic treatment for esophageal achalasia. Both endoscopic and surgical treatment options are available for the treatment of esophageal achalasia.^13)^ However, once a perforation has occurred, additional endoscopic treatment for esophageal achalasia becomes difficult, and surgical intervention is necessary for further management. Although the perforation was localized to the mediastinum, episodes of hypoxemia and tachycardia indicated the need for surgery. Moreover, selecting conservative management could potentially negatively impact future surgical interventions for achalasia. The patient’s general condition was not critically compromised, which permitted a relatively prolonged operative time. Considering the abovementioned factors, a minimally invasive surgery was selected. The following were the surgical options considered: 1) staged closure of the perforation site followed by treatment for esophageal achalasia; 2) performing the previously mentioned procedure in a single stage; and 3) performing esophagectomy with gastric reconstruction. We opted for a two-stage approach, considering the patient’s stable preoperative condition and the relatively clean perforation site observed intraoperatively. Ultimately, the selected surgical intervention was performed as described, resulting in no postoperative complications and facilitating a relatively early discharge after 22 days of hospitalization. Among the various treatment strategies, the ability to simultaneously perform emergency surgery for esophageal perforation and radical treatment for esophageal achalasia constitutes a significant positive aspect of this case. Additionally, treatment was achieved without causing severe adverse events to the patient and led to a relatively short hospital stay.

## CONCLUSIONS

We reported a case wherein a patient underwent staged laparoscopic surgery for esophageal perforation following PD for esophageal achalasia. The current case highlighted successful treatment of esophageal perforation with a minimally invasive surgery, and esophageal achalasia was managed using the laparoscopic Heller–Dor procedure. Both the procedures were performed with minimal invasiveness, achieving curative results within a relatively short period.

## SUPPLEMENTARY MATERIALS

Supplementary Fig. 1

## DECLARATIONS

### Availability of data and materials

The datasets generated and analyzed during the current study are available from the corresponding author upon reasonable request.
