# Supplementary material for: Esophageal Perforation Following Pneumatic Dilation in Esophageal Achalasia Successfully Managed with Two-Stage Laparoscopic Surgery
Source: Surg Case Rep. 2025 Nov 29;11(1):25-0418. doi: 10.70352/scrj.cr.25-0418 (PMC12668788; doi:10.70352/scrj.cr.25-0418)
Supplement: Supplementary Fig. 1 [file scr-11-01-25-0418-s001.pdf]

Supplementary Figure1

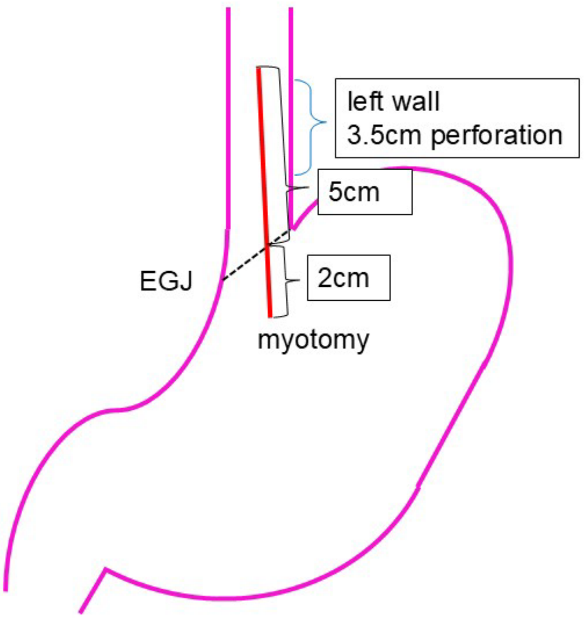

Supplementary Fig. 1　　Relationship between the esophageal perforation and the myotomy site
